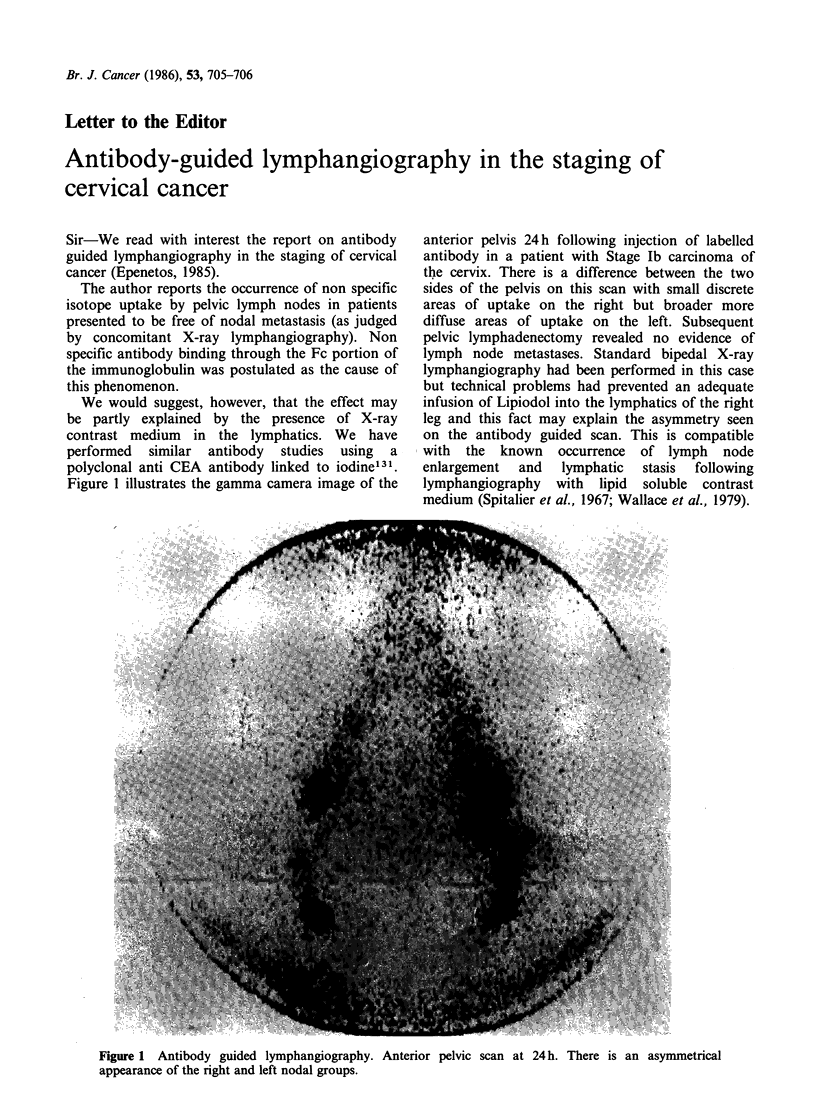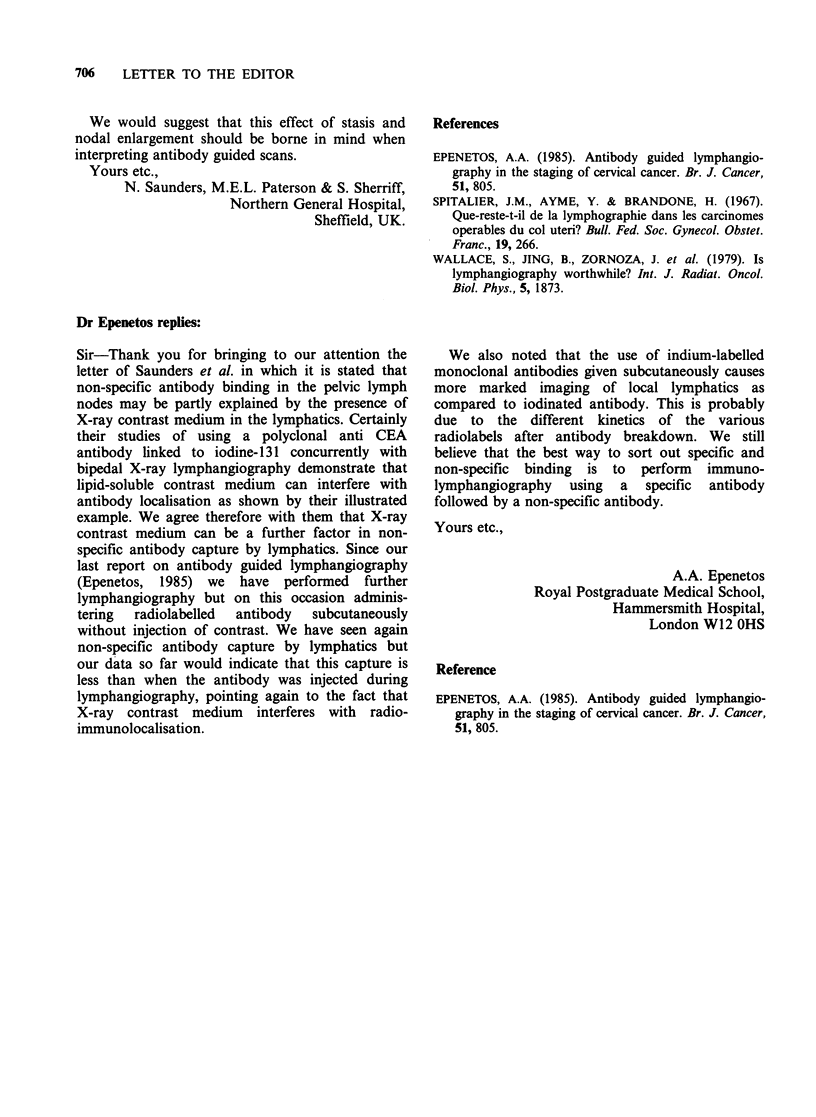# Antibody-guided lymphangiography in the staging of cervical cancer.

**DOI:** 10.1038/bjc.1986.118

**Published:** 1986-05

**Authors:** N. Saunders, M. E. Paterson, S. Sherriff

## Abstract

**Images:**


*Br. J. Cancer* (1986), **53**, 705–706

### Letter to the Editor

### Antibody-guided lymphangiography in the staging of cervical cancer

Sir—We read with interest the report on antibody guided lymphangiography in the staging of cervical cancer (Epenetos, 1985).

The author reports the occurrence of non specific isotope uptake by pelvic lymph nodes in patients presented to be free of nodal metastasis (as judged by concomitant X-ray lymphangiography). Non specific antibody binding through the Fc portion of the immunoglobulin was postulated as the cause of this phenomenon.

We would suggest, however, that the effect may be partly explained by the presence of X-ray contrast medium in the lymphatics. We have performed similar antibody studies using a polyclonal anti CEA antibody linked to iodine$^{131}$. Figure 1 illustrates the gamma camera image of the anterior pelvis 24 h following injection of labelled antibody in a patient with Stage Ib carcinoma of the cervix. There is a difference between the two sides of the pelvis on this scan with small discrete areas of uptake on the right but broader more diffuse areas of uptake on the left. Subsequent pelvic lymphadenectomy revealed no evidence of lymph node metastases. Standard bipedal X-ray lymphangiography had been performed in this case but technical problems had prevented an adequate infusion of Lipiodol into the lymphatics of the right leg and this fact may explain the asymmetry seen on the antibody guided scan. This is compatible with the known occurrence of lymph node enlargement and lymphatic stasis following lymphangiography with lipid soluble contrast medium (Spitalier *et al.*, 1967; Wallace *et al.*, 1979).

**Figure 1** Antibody guided lymphangiography. Anterior pelvic scan at 24 h. There is an asymmetrical appearance of the right and left nodal groups.

We would suggest that this effect of stasis and nodal enlargement should be borne in mind when interpreting antibody guided scans.

Yours etc.,

N. Saunders, M.E.L. Paterson & S. Sherriff,
Northern General Hospital,
Sheffield, UK.

### References

EPENETOS, A.A. (1985). Antibody guided lymphangiography in the staging of cervical cancer. *Br. J. Cancer*, **51**, 805.

SPITALIER, J.M., AYME, Y. & BRANDONE, H. (1967). Que-reste-t-il de la lymphographie dans les carcinomes operables du col uteri? *Bull. Fed. Soc. Gynecol. Obstet. Franc.*, **19**, 266.

WALLACE, S., JING, B., ZORNOZA, J. *et al.* (1979). Is lymphangiography worthwhile? *Int. J. Radiat. Oncol. Biol. Phys.*, **5**, 1873.